# Supplementary material for: Applications of Genome-Wide Screening and Systems Biology Approaches in Drug Repositioning
Source: Cancers (Basel). 2020 Sep 21;12(9):2694. doi: 10.3390/cancers12092694 (PMC7563533; doi:10.3390/cancers12092694)
Supplement: Supplementary file 1 [file cancers-12-02694-s001.pdf]

Supplementary Materials: Applications of Genome-Wide Screening and Systems Biology Approaches in Drug Repositioning

Elyas Mohammadi, Rui Benfeitas, Hasan Turkez, Jan Boren, Jens Nielsen, Mathias Uhlen and Adil Mardinoglu

Table S1. An overview of all DBs discussed in this review in case of type of assay(s), data and availability of content.

Database	Type of data	Assays	Chemical content	Biological content	Public Av.	Ref
METN	Lipids, amino acids, carbohydrates, toxins, small peptides, and natural products, among other classes	High-resolution tandem mass spectrometry	-	64,000 structures metabolite structures	Y	[1]
PACO	Biochemical reactions, assembly of biomolecular complexes, transport and catalysis events and physical interactions	Data integration	-	4794 detailed human biochemical processes (i.e. pathways) and ~2.3 million interactions	Y	[2]
L1000	Gene expression profiles for thousands of perturbagens at a variety of time points, doses, and cell lines	L1000 mRNA profiling assay	32,855 small molecules,	1.3M L1000 profiles, 476251 expression signatures, 99 Cell line, 7 datasets, 6 doses, 2 time points (24 h, 3 h),	Y	[3,4]
DPTH	Drug-induced pathways	KEGG pathway enrichment analysis	1294 drugs	243,272 drug-pathway pairs, and 94 KEGG pathways	Y	[5]
PRIDE	The world’s largest data repository of mass spectrometry-based proteomics data	Data collection	-	A living repository which has daily update	y	[6]
CORUM	Protein complexes	manually curation	-	4274 protein complexes, 4473 different genes	Y	[7]
SAINT	Protein-protein interaction	Statistical model		A computational tool assigning confidence scores to protein-protein interactions based on affinity purification-mass spectrometry (AP-MS) data	Y	[8]
CTD	Chemical–gene/protein interactions, chemical–disease and gene–disease relationships	manually curation	15681 chemicals	44,366,867 toxicogenomic relationships, 46 689 genes, 4340 phenotypes and 7212 diseases	Y	[9]
Entrez	nucleotide and protein sequence data, gene-centered and genomic mapping information, 3D structure data, PubMed MEDLINE etc.	Data collection from other datasets and data integration	-	A living repository which has daily update	Y	[10]

Ensemble	Genomic data	Aggregating, processing, integrating and redistributing genomic datasets	-	A living repository which has daily update	Y	[11]
GEO						
DSDB	drug and small molecule related gene sets	Gene set enrichment analysis	17,389 Unique compounds	22,527 Gene sets, 19 531 genes	Y	[12]
HIVRT	Protease, Reverse Transcriptase, and integrase sequences or mutations	Meta-analysis	-	Sequences from 715 protease inhibitors, 585 nucleoside reverse transcriptase inhibitors; and 296 integrase inhibitors	Y	[13]
TDR	Genomic data	Integrating genomic data	2.0 M Bioactive compounds	5.3 K Druggable Targets, 7.2 M Bioactivities, 45 Full Proteomes, 20 Genome-wide prioritizations, 1.2 M Annotations	Y	[14]
SBIOS	Molecular replacements and their performance in biochemical assays	Detection of matched molecular pairs and mining bioactivity data in the ChEMBL database	-	21,293,355 datapoints corresponding to 5,586,462 unique replacements that have been measured in 35,039 assays against 1948 molecular targets representing 30 target classes	Y	[15]
DMC	information on active ingredients chemical entities, pharmaceutical products, drug mode of action, indications, pharmacologic action	Data integration	4444 Active ingredients, 2021 FDA drugs, 2423 drugs approved outside US, 3799 small molecules etc.	239 Biologics and peptides, 600 human protein targets , 194 infectious agents targets, 43 metabolites and biopolymers, 1500 protein–drug (and vice versa) crystal complex (PDB), 13825 bioactivity data points, 10427 human proteins	Y	[16]
DRAR	Drug molecules and targetable human proteins	Docking score	254 Active forms of 166 small molecules	385 Pocket models of 353 proteins with known functions,	Y	[17]
SCYP	Interactions of phase I and II enzymes and drug transporters with drugs, prodrugs, alimentary and Traditional Chinese Medicine compounds	Data integration, text mining and manual validation	3000 Drugs	>350 relevant food ingredients (e.g. grapefruit juice) and herbs, which are catalyzed by 400 proteins, 10, 000 interactions, 200 3D structures of relevant proteins	Y	[18]

CTDB	A tool to predict chemical hazards	Data curation	70 Million structures 80,908 chemicals with 833,844 labeled hazard endpoints	70 Million structures	Y	https://www.ul.com/
PDTD	Protein, diseases, biological functions and pathways.	Using TarFisDock, a web server tool, for Data integration and target identification	-	>1100 protein entries with 3D structures, >830 known or potential drug targets	Y	[19]
ODB	Recipients, payment, claims, and pharmacy and practitioner information	Manually collection	-	-	N	[20]
DTW	A web-based interface	*DT*-Hybrid algorithm, domain-specific knowledge expressing drugs, targets similarity	Drugs extracted from DrugBank database	-	Y	[21]
DNET	Disease relationships, pathways, genes	Differential co-expression analysis	342 Disease related drugs	5598 pathways, 7357 disease-related genes	Y	[22]
ARREXP	Functional Genomics Data	High-throughput functional genomics experiments	-	73,024 Experiments, 2,436,379 assays, and 56.67 TB of archived data	Y	[23]
BLGe	Genes related to protein kinases, ion channels and nuclear receptors	Text mining and a bibliometric analysis on kinases, ion channels and nuclear receptors.	-	300 Genes	Y	[24]
RLa	Druggable genes	Text mining	-	3361 Genes	Y	[25]
MskIm	Somatic mutations	Memorial Sloan Kettering-Integrated Mutation Profiling of Actionable Cancer Targets (MSK-IMPACT)	-	341 key cancer genes	Y	[26]
HkG	Druggable genes	Text mining	-	2676 Genes	Y	[27]
HCa	Druggable human proteins	Text mining		4479 Genes	Y	[28]

GTPGe	Pharmacological, chemical, genetic, functional and pathophysiological data on the targets of approved and experimental drugs	Data curation and integration of	-	1969 Genes	Y	[29]
GO	Biological process, molecular function and cellular component	Computational model of biological systems	-	7,728,430 Number of annotations, 3,007,085 Annotations for biological process, 2,388,698 Annotations for molecular function, 2,332,647 Annotations for cellular component, etc.	Y	[30]
FOGe	Tumor genomic alterations	Targeted, massively parallel sequencing approach	-	243 Gene	Y	[31]
dGene	An annotation tool designed to identify druggable genes	Data curation and annotation	-	2257 Genes	Y	[32]
CMI	Personalize treatment options	Many assays including Tumor Profiling and tumor Mutational Burde	-	66 Genes	Y	https://www.carislifesciences.com/
TTD	Therapeutic targets and corresponding drugs	Text mining, BLAST, Tanimoto similarity searching	5028 drugs	1894 targets, 560 diseases and, 3049 interactions	Y	[33]
TEND	Drug targets, drugs, interactions	Manual curation	989 Drugs	437 Gene targets, 2243 interactions	Y	[34]
TDGCT	Clinical trial drug-target interactions	Manual curation	2419 Drugs	1016 Genes, 5063 interactions	Y	[35]
TALC	Targeted therapies	Manual collection	274 Drugs	191 Genes, 624 interactions	Y	[36]
PhGKB	Clinical pharmacogenomic biomarkers	Manual curation	589 Drugs	600 Genes, 1952 interactions	Y	[37]
OncoKB	Unique mutations, fusions, and copy number alterations	Annotation	99 Drugs	50 Genes, 189 interactions	Y	[38]
NCI	Various cancer types and related information	Cancer biology, cancer genomics, causes of cancer, diagnosis prevention screening, early detection, and treatment assays	1519 Drugs	1045 Genes, 6231 interactions	Y	https://wiki.nci.nih.gov/display/cageneindex
MCGCT	Clinical trials, biomarkers, pathways, disease, and drugs	Various assays related to biomarker identification	252 Drugs	182 Genes, 234 interaction	Y	[39]

GPIs	Pharmacological, chemical, genetic, functional and pathophysiological data	Data curation and integration of	6495 Drugs	1608 Genes, 12593 interactions	Y	[29]
GRNAi	RNAi screening data	text-mining	-	127 screens in human, and 170 in *Drosophila*, 53 of which have been performed in vivo, 500,000 phenotypes.	Y	[40]
Rep DB	Disease, repurposed drugs and drug targets	Literature, Enrichment analysis of current repositioned drugs	187 small molecules	1125 Diseases, 64 protein drugs	Y	[41]
NCATS	Translational data	Quantitative High-Throughput Screening	2900 drugs [small molecules]	-	N	https://ncats.nih.gov/
Encode	Chromatin, histone mark enrichment, transcription factor binding, gene expression, 3D chromatin interactions.	DNase-seq, ATAC-seq, ChIP-seq, TF ChIP-seq, RNA-seq, ChIA-PET		Omics data from 13,393 biosamples	Y	[42]
CTR	Gene targets, Protein targets, Compound structures,	Quantitative lineage tracing	481 small-molecule probes,	860 deeply characterized cancer-cell lines, eight doses	Y	[43]
CCLE	genomic data, analysis and visualization for 1457 cell lines	RNA whole-exome, whole-genome, reduced representation bisulfite sequencing, reverse-phase protein array, microRNA expression and global histone modification profiling	-	1457 Cell Lines, 84,434 genes, 136488 unique Data Sets, 1,159,663 mutation Entries	Y	[44]
GDSC	mRNA expression, Cell Line Annotations, Oncomap mutations, Hybrid capture sequencing, Pharmacological Profiling	Resazurin or Syto60, Cell Titre-Glo	565 compounds,	1796 cell lines, 446,146 IC50s	Y	[45]
GPCR	Drug library	GPCR assay	398,449 compounds	-	Y	[46]

DRUGB	sequence, structure, and pathway of drug targets, chemical, pharmacological and pharmaceutical data of drugs	pharmacoproteomic s, pharmacotranscripto mics	13,529 drugs	5200 non-redundant protein (i.e. drug target/enzyme/transporter/carrier)	*Y*	[47]
OGEE	Genes and expression profiles, duplication status, conservation across species, evolutionary origins	Text-mining	-	99 Gene essentiality experiments for 9 eukaryotes and 39 prokaryotes	*Y*	[48]
DRHUB	Multi omics	Integration of multiple public sources	6125 unique compounds, 663 drug indications	10,147 samples, 2247 protein targets,	*Y*	[49]
CCLF	Cancer cell line models	Tube barcoding, Molecular fingerprinting, patient-derived xenografts and etc.	-	100 new Cancer cell line models	*Y*	[50]
ACHI	Essential genes	Genome-scale RNAi, CRISPR-Cas9 loss-of-function screening, Computational Modeling	-	~2000 cell lines	*N*	[51]
DMAP	Computational models of vulnerabilities	Collecting data from subdivisions of the DMAP project though predictive modeling	4686 Drug sensitivity	CRISPR (18,333 genes), 1775 cell Line sample info, 19144 expression, 18,802 mutation, 214 Protein Array, 17,309 Combined RNAi	*Y*	[51]
DGIdb	Gene, drug, Gene-Drug interaction	Text mining and manually curation of data	22,342 drugs,	99,404 genes, 55,702 Gen-Drug interactions	*Y*	[52]
DSIG	Drugs, gene targets	Literature review, data collection from other DBs	>1300 drugs,	7000 microarray and 800 gene targets	*Y*	[12]
HTRCP	Transcription profiles	Literature review	-	50 clinically important human pathogens, 1353 gene-expression profiles generated from >60 human cells/tissues	*Y*	[53]

CTRP	Protocol information, Consistent terminology and coding to parse cancer trials information	Clinical trials	-	A collection of NCI-supported interventional clinical trial data	*Y*	https://www.cancer.gov/about-nci/organization/ccct/ctrp
PBL	Accepts protein structures	Ligand-Protein binding prediction tool	-	-	*Y*	[54]
CCOM	A Web - based platform that records the relevant genomic and clinical data from patients, produces actionable information regarding potential treatments (including applicable clinical trials), captures outcomes data, and makes these data available for researchers to improve knowledge about the disease.	CCOM platform	-	-	*N*	[55]
CGI	Cancer biomarkers, oncogenic mutations, cancer genes, cancer bioactivities	Literature review	1631 biomarkers of drug response,	5601 validated oncogenic alterations, 765 cancer genes, 246 cancer types	*Y*	https://www.cancergenomeinterpreter.org/
ChBLI	Chemical, bioactivity and genomic data	Text mining and manually curation of data	13,377 Targets,	76,076 Publications, and 57 Deposited Datasets	*Y*	[56]
CIViC	Genes, Variants, Evidence Items, Assertions, Disease, Variant, and Clinical Significance	Text mining and manually curation of data	>25 Drugs	~7500 evidences, >25 Disease,	Y	[57]
CKB	gene, gene variants, drug, drug class, indication, and clinical trials	Text mining and manually curation of data	-	358-gene panel, 82 common driver genes in cancer	Y	[58]
CFBs, CFCT	Protein biomarker expression, Gene abnormalities	Molecular tumor profiling	179 Drug	144 Gene, 344 interaction	Y	http://www.clearityfoundation.org
DoCM	Gene variants	Text mining and manually curation of data	-	1,364 variants from 876 unique publications	Y	[59]

FDA	Biomarkers, Pharmacogenomic information	Drug labeling	204 Drug	404 Biomarkers, 62 Genes, 270 interaction	Y	https://www.fda.gov/drugs/scienceresearch/researchareas/pharmacogenetics/ucm083378.htm
SMPDB	Small Molecule Pathways	Text mining and manually curation of data	696 drugs,	48,690 pathways, 55,700 metabolites, 1451 proteins, 791 enzymes, 137 transporters, 57402 reactions, 294 transportations, 691 Interactions	Y	[60]
GSEA/ MSigDB	Annotated gene sets	Gene Set Enrichment Analysis	-	10,925 gene sets	Y	[61]
PROM	Protein-protein and drug-protein interactions	Text and data mining including manual curation	25,000 Drugs	21,500 Drug-protein, 104,000 protein-protein interaction	Y	[62]
BMRB	Quantitative NMR spectral parameters for proteins, peptides, nucleic acids, carbohydrates and ligands or cofactors	NMR	-	250 Metabolites, over 4500 entries containing NMR spectral values and derived information	Y	[63]
REMC	Human epigenomic data	Next-generation sequencing technologies	-	2804 Genome-wide datasets, including 1821 histone modification datasets, 360 DNase datasets, 277 DNA methylation datasets, and 166 RNA-Seq datasets.	Y	[64]
GSDB	Diverse biological information with a particular focus on human disease and pharmacology	Data integration, gene set enrichment		Integrates 26 databases containing different types of information	Y	[65]

Public Av.: Public availability; Y: Yes; N: No.

© 2020 by the authors. Licensee MDPI, Basel, Switzerland. This article is an open access article distributed under the terms and conditions of the Creative Commons Attribution (CC BY) license (http://creativecommons.org/licenses/by/4.0/).
